# Supplementary material for: Modeling glioblastoma heterogeneity as a dynamic network of cell states
Source: Mol Syst Biol. 2021 Sep 16;17(9):e10105. doi: 10.15252/msb.202010105 (PMC8444284; doi:10.15252/msb.202010105)
Supplement: Supplementary file 6 — Source Data for Figure 5 [file MSB-17-e10105-s004.zip › Figure5A_sourcedata/GSEA_3071/hallmarks_stateVI.GseaPreranked.1623742557031/HALLMARK_COMPLEMENT.html]

Details for gene set HALLMARK\_COMPLEMENT[GSEA]

|  || Dataset | state63071 |
| Phenotype | NoPhenotypeAvailable |
| Upregulated in class | na\_neg |
| GeneSet | HALLMARK\_COMPLEMENT |
| Enrichment Score (ES) | -0.26770613 |
| Normalized Enrichment Score (NES) | -1.1576045 |
| Nominal p-value | 0.29638556 |
| FDR q-value | 0.55971575 |
| FWER p-Value | 1.0 |
Table: GSEA Results Summary

  

Fig 1: Enrichment plot: HALLMARK\_COMPLEMENT      
 Profile of the Running ES Score & Positions of GeneSet Members on the Rank Ordered List

  

| PROBE | GENE SYMBOL | GENE\_TITLE | RANK IN GENE LIST | RANK METRIC SCORE | RUNNING ES | CORE ENRICHMENT || 1 | SERPINE1 |  |  | 0 | 1.673 | 0.0611 | No |
| 2 | F3 |  |  | 26 | 0.988 | 0.0902 | No |
| 3 | PLAUR |  |  | 34 | 0.936 | 0.1224 | No |
| 4 | GATA3 |  |  | 36 | 0.929 | 0.1560 | No |
| 5 | DUSP5 |  |  | 169 | 0.631 | 0.1424 | No |
| 6 | MAFF |  |  | 253 | 0.562 | 0.1397 | No |
| 7 | PFN1 |  |  | 347 | 0.513 | 0.1326 | No |
| 8 | HSPA5 |  |  | 414 | 0.484 | 0.1319 | No |
| 9 | TNFAIP3 |  |  | 445 | 0.473 | 0.1408 | No |
| 10 | CD55 |  |  | 452 | 0.471 | 0.1563 | No |
| 11 | EHD1 |  |  | 455 | 0.469 | 0.1729 | No |
| 12 | CTSB |  |  | 471 | 0.464 | 0.1857 | No |
| 13 | PIM1 |  |  | 582 | 0.430 | 0.1707 | No |
| 14 | CSRP1 |  |  | 671 | 0.407 | 0.1611 | No |
| 15 | TFPI2 |  |  | 727 | 0.391 | 0.1601 | No |
| 16 | CTSL |  |  | 767 | 0.383 | 0.1632 | No |
| 17 | IRF1 |  |  | 771 | 0.382 | 0.1763 | No |
| 18 | ME1 |  |  | 818 | 0.374 | 0.1771 | No |
| 19 | DOCK10 |  |  | 858 | 0.367 | 0.1797 | No |
| 20 | PCLO |  |  | 985 | 0.348 | 0.1573 | No |
| 21 | PRDM4 |  |  | 1125 | 0.327 | 0.1306 | No |
| 22 | RHOG |  |  | 1355 | 0.297 | 0.0776 | No |
| 23 | CEBPB |  |  | 1389 | 0.293 | 0.0791 | No |
| 24 | FN1 |  |  | 1654 | 0.264 | 0.0153 | No |
| 25 | CTSC |  |  | 1767 | 0.254 | -0.0066 | No |
| 26 | CTSD |  |  | 1862 | -0.259 | -0.0233 | No |
| 27 | CTSO |  |  | 2060 | -0.288 | -0.0676 | No |
| 28 | FYN |  |  | 2118 | -0.301 | -0.0725 | No |
| 29 | CD46 |  |  | 2310 | -0.335 | -0.1134 | No |
| 30 | C1R |  |  | 2388 | -0.351 | -0.1220 | No |
| 31 | MMP14 |  |  | 2440 | -0.365 | -0.1229 | No |
| 32 | KIF2A |  |  | 2532 | -0.385 | -0.1342 | No |
| 33 | CASP3 |  |  | 2699 | -0.426 | -0.1649 | No |
| 34 | CALM1 |  |  | 2784 | -0.451 | -0.1718 | No |
| 35 | GNG2 |  |  | 2802 | -0.459 | -0.1598 | No |
| 36 | TIMP2 |  |  | 3180 | -0.643 | -0.2412 | Yes |
| 37 | GNAI2 |  |  | 3183 | -0.647 | -0.2182 | Yes |
| 38 | PDP1 |  |  | 3362 | -0.826 | -0.2376 | Yes |
| 39 | ZEB1 |  |  | 3363 | -0.827 | -0.2074 | Yes |
| 40 | GNB4 |  |  | 3390 | -0.874 | -0.1827 | Yes |
| 41 | CDH13 |  |  | 3410 | -0.913 | -0.1547 | Yes |
| 42 | PLAT |  |  | 3416 | -0.927 | -0.1222 | Yes |
| 43 | PRCP |  |  | 3417 | -0.927 | -0.0884 | Yes |
| 44 | PDGFB |  |  | 3506 | -1.131 | -0.0716 | Yes |
| 45 | DUSP6 |  |  | 3512 | -1.151 | -0.0310 | Yes |
| 46 | CLU |  |  | 3601 | -1.801 | 0.0103 | Yes |
Table: GSEA details [plain text format]

  

Fig 2: HALLMARK\_COMPLEMENT: Random ES distribution      
 Gene set null distribution of ES for **HALLMARK\_COMPLEMENT**
